# Retraction: *Astragali radix* isoflavones synergistically alleviate cerebral ischemia and reperfusion injury via activating estrogen receptor-PI3K-Akt signaling pathway

**DOI:** 10.3389/fphar.2023.1252192

**Published:** 2023-07-06

**Authors:** Frontiers Editorial Office

Following publication, concerns were raised regarding the integrity of the images in the published figures. The authors failed to provide a satisfactory explanation during the investigation, which was conducted in accordance with Frontiers’ policies.

The authors agree to this retraction.

This retraction was approved by the Chief Editors of Frontiers in Pharmacology and the Chief Executive Editor of Frontiers.

